# Genetic factors involves in intracranial aneurysms – actualities

**Published:** 2015

**Authors:** D Mohan, V Munteanu, T Coman, AV Ciurea

**Affiliations:** * Department of Neurosurgery, Clinical Emergency Hospital Oradea, Romania; ** Department of Neurosurgery, “Sanador” Medical Center Hospital, Bucharest, Romania; *** Department of Neurosurgery, “Bagdasar-Arseni” Clinical Hospital, Bucharest, Romania; **** Romanian Ministry of Health Neurosurgical Committee; “Sanador” Medical Center Hospital, Bucharest, Romania; “Carol Davila” University of Medicine and Pharmacy, Bucharest, Romania

**Keywords:** Intracranial aneurysm, genetic, chromosome, factor

## Abstract

Intracranial aneurysm (IA) is a common vascular disorder, which frequently leads to fatal vascular rupture leading to subarachnoid hemorrhage (SAH). Although various acquired risk factors associated with IAs have been identified, heritable conditions are associated with IAs formation but these syndromes account for less than 1% of all IAs in the population. Cerebral aneurysm disease is related to hemodynamic and genetic factors, associated with structural weakness in the arterial wall, which was acquired by a specific, often unknown, event. Possibly, the trigger moment of aneurysm formation may depend on the dynamic arterial growth, which is closely related to aging/ atherosclerosis. Genetic factors are known to have an important role in IA pathogenesis. Literature data provide complementary evidence that the variants on chromosomes 8q and 9p are associated with IA and that the risk of IA in patients with these variants is greatly increased with cigarette smoking. Intracranial aneurysms are acquired lesions (5-10% of the population). In comparison with sporadic aneurysms, familial aneurysms tend to be larger, more often located in the middle cerebral artery, and more likely to be multiple.

**Abbreviations:** DNA = deoxyribonucleic acid, FIA = familial Intracranial Aneurysm, GWAS = genome-wide association studies, IL-6 = interleukin-6, ISUIA = International Study of Unruptured Intracranial Aneurysms, IA = Intracranial aneurysm, mRNA = Messager ribonucleic acid, SNPs = single-nucleotide polymorphisms, SMCs = smooth muscle cells, sIAs = sporadic IAs, SAH = subarachnoid hemorrhage, TNF-α = tumor necrosis factor-alpha, COL4A1 = type IV collagen alpha-1

## Introduction

Intracranial aneurysm (IA) is a common vascular disorder, which frequently leads to fatal vascular rupture leading to subarachnoid hemorrhage (SAH). Although various acquired risk factors associated with IAs have been identified, heritable conditions are associated with IAs formation but these syndromes account for less than 1% of all IAs in the population [**3**]. Rupture of an IA leads to a SAH, which is fatal in about 50% of the cases. IAs are relatively common with an estimated prevalence of unruptured IAs of 2%-6% in the adult population, and are considered a complex disease with both genetic and environmental risk factors. Known risk factors include smoking, hypertension, increasing age, and positive family history. Identifying the molecular mechanisms underlying the pathogenesis of IAs is complex. Genome-wide approaches such as DNA linkage and genetic association studies, as well as microarray-based mRNA expression studies, provide approaches to identify genetic risk factors and dissecting the molecular pathobiology of IAs [**36**].

## Risk factors

Cerebral aneurysm disease is related to hemodynamic and genetic factors, associated with structural weakness in the arterial wall, which was acquired by a specific, often unknown, event. Possibly, the trigger moment of aneurysm formation may depend on the dynamic arterial growth, which is closely related to aging/ atherosclerosis [**37**]. Risk factors also differ according to the site of aneurysm. A prospectively collected database study of patients with aneurysmal SAH in patients with saccular IAs relived that in comparison with aneurysms at the anterior communicating artery, those at the middle cerebral artery, were less associated with age >55, those at the posterior communicating artery were less associated with male gender and those at the basilar artery were more associated with no alcohol consumption [**21**]. To define the characteristics of familial IAs in comparison with the characteristics of nonfamilial IAs, FIA and ISUIA study were compared. The Familial Intracranial Aneurysm (FIA) study is a multicenter international study with the goal of identifying genetic and other risk factors for formation and rupture of IAs in a highly enriched population. International Study of Unruptured Intracranial Aneurysms (ISUIA) regard to patient demographic data, IA location, and IA multiplicity. The author’s conclusion was that multiplicity was more common in the FIA patients (35.6% vs. 27.9%) and the FIA patients had a higher proportion of IAs located in the middle cerebral artery (28.6% vs. 24.9%), whereas ISUIA patients had a higher proportion of posterior communicating artery IAs (13.7% vs. 8.2%) [**24**]. 

The risk of aneurysm is increased by a family history of aneurysms, and in the middle of certain populations, specifically in Japan and Finland. Several other risk factors were documented, including hypertension, smoking, alcohol consumption, and female sex. Estrogen protects several components within the artery wall, and inhibits some of the inflammatory molecules that could cause aneurysms. At menopause, the estrogen level decreases and the incidence of aneurysm increases. Hemodynamic stresses have been shown to be involved in the formation, growth and rupture of aneurysms. This is often associated with hypertension, which also increases the risk of aneurysm rupture [**9**]. 

## Experimental studies

The transcription factor Sox17 is robustly expressed in endothelial cells of normal intracerebral arteries. The combination of Sox17 deficiency and angiotensin II infusion in mice induces vascular abnormalities like luminal dilation, wall thinning, tortuosity, and SAH. Furthermore, human IA samples showed reduced Sox17 expression and decreased endothelial integrity. A recent study demonstrated that Sox17 deficiency in mouse could induce IA under hypertensive conditions, suggesting Sox17 deficiency as a potential genetic factor for IA formation [**18**]. 

## Genetic studies

Genetic factors are known to have an important role in IA pathogenesis. Literature data provide complementary evidence that the variants on chromosomes 8q and 9p are associated with IA and that the risk of IA in patients with these variants is greatly increased with cigarette smoking [**6**]. A study identified chromosomal regions on chromosomes 4, 7, 8, and 12 likely to harbor genes that contribute to the risk of smoking in IA [**8**]. Significant evidence of linkage to IA was also found on chromosome 8p22.2 [**15**]. A new IA susceptibility locus on 13q was identified [**34**]. Genome-wide association studies, which contribute to IA susceptibility, confirmed an association in a sample on chromosome 9, in a gene previously associated with IA and novel region on chromosome 7 - which has previously been associated with ischemic stroke and the large vessel stroke occlusive subtype, suggesting a possible genetic link between this stroke subtype and IA [**7**]. 

Genetic in IA varied also by ethnic factors. About 3% of the population develops saccular intracranial aneurysms (sIAs), however, the sIA-SAH incidence in Finland is >2× increased. A recent analysis compared the frequencies and effect sizes of the replicated variants in Finnish sIA patients (developed from familial sIA), and in European population and relived four new high-risk loci with low frequency lead variants. Three were associated with the case-control status: 2q23.3; 5q31.3; 6q24.2 and one with the number of sIAs: 7p22.1. The 7p22.1 locus was strongly differentiated and was more frequent in Finland (4.6%) than in the Netherlands (0.3%). Additionally, a previously inconclusive locus on 2q33.1 was replicated. The five loci explain 2.1% of the sIAs heritability in Finland [**17**]. The genome-wide association studies (GWAS) have identified five loci with strong association and further 14 loci with suggestive association with IA. The blood pressure (BP) is a strong risk factor of IA. The suggestive IA locus at 5q23.2 was significantly associated with SBP in individuals of European descent [**11**]. In a Japanese population using allelic and haplotype association analyses, only one variant was found, rs767603, at chromosome 14q23, which was significantly associated with IA, both in allelic analysis and haplotype analysis [**26**]. A study on a large cohort made to identify single-nucleotide polymorphisms (SNPs) that are associated with IAs in Japanese population, relived that 45 SNPs in 24 genes encoding proteins have been considered possible risk factors to IAs pathogenesis [**23**]. Another study investigated whether risk alleles of single nucleotide polymorphisms associated with IA are developed in patients with familial IA, IA located at the middle cerebral artery, or IA rupture at a younger age. Authors calculated the genetic risk scores for 973 Dutch and 718 Finnish patients with IA by summing the effect size-weighted risk allele counts of 7 SNPs, associated with IAs previously identified through genome-wide association studies and found that genetic risk factors have a larger role in the development of IA at the middle cerebral artery than at other sites, and that genetic heterogeneity should be considered in future genetic studies [**38**]. Genetic risk factors for IAs may influence the size of aneurysms. Genotypes of 7 independent SNPs of the 6 genetic risk loci identified in genome-wide association studies of Dutch patients with IAs relived that the association between SNPs and size was assessed for single SNPs and for the combined effect of SNPs by using a weighted genetic risk score. Single SNPs showed no association with aneurysm size, nor did the genetic risk score. The 6 genetic risk loci have no major influence on the size of aneurysms at the time of rupture. Because these risk loci explain no more than 5% of the genetic risk, other genetic factors for IAs may influence the aneurysm size and so lead to rupture [**16**]. Recently, genome wide association studies have identified the 9p21 region as a risk locus for IA in a Swedish population. There is one study showing an association between 9p21 and arterial stiffness, and arterial stiffness plays a role in the development of hypertension [**28**]. In order to identify the risk factors for sporadic intracranial aneurysm (IA) development and rupture, authors identified the 19 SNPs associated with IA. The strongest associations, robust to sensitivity analyses for statistical heterogeneity and ethnicity, were found for the following SNPs: on chromosome 9 within the cyclin-dependent kinase inhibitor 2B antisense inhibitor gene, on chromosome 8 near the SOX17 transcription regulator gene, and on chromosome 4 near the endothelin receptor A gene [**1**].

Familial occurrence of IAs suggests that there are 2 potential loci in the Dutch family, on chromosome regions 1p36 and Xp22. Additional microsatellite markers were genotyped in the 2 candidate loci and showed suggestive linkage to the locus on chromosome 1 with a nonparametric linkage of 3.18 at 1p36.11-p36.13 and significant linkage to the locus on chromosome X with a nonparametric linkage of 4.54 at Xp22.2-p22.32 [**33**]. Another analysis identified 10 genome-wide linkage in families and sib pairs with IA. These studies have identified several loci, but only 4 (1p34.3-p36.13, 7q11, 19q13.3, and Xp22) have been replicated in different populations. For the loci on 1p34.3-p36.13 and 7q11, the association with the positional candidate genes has also been demonstrated: for locus on 1p34.3-p36.13 association with the perlecan gene and for 7q11 association with the elastin and collagen type 1 A2 genes [**32**]. 

A recent genome-wide association study of IAs in Finnish, Dutch and Japanese cohorts totaling 5,891 cases and 14,181 controls, identified three new loci strongly associated with IAs on chromosomes 18q11.2 and 10q24.32, and replicated two previously found loci on chromosomes 8q11.23-q12.1 and 9p21.3. However, these five IAs risk loci identified so far explain only up to 5% of the familial risk of IAs, which makes genetic risk prediction tests currently unfeasible [**31**]. Another genome-wide association study identified associations between SNPs on chromosome 9p21 and risk of harboring IAs. Aneurysm characteristics or subphenotypes of IAs, such as history of SHA, presence of multiple IAs and location of IAs, are clinically important. Among the most common sites of IAs, the association was the strongest for IAs of the posterior communicating artery and not significant for IAs in the anterior communicating artery. When dichotomizing IA sites, the association was stronger for IAs of the posterior circulation-posterior communicating artery group vs. the anterior circulation group [**27**]. 

## Smooth muscle cells and the formation, degeneration, and rupture of saccular intracranial aneurysm wall

Around 3.5% of the middle-aged otherwise healthy population carries unruptured sporadic IAs (sIAs). Many sIAs never rupture. Smooth muscle cells (SMCs) play a critical role both in the formation of sIAs, as well as in the repair and adaptation of the sIA wall to hemodynamic and proteolytic stress to which it is subjected. Loss of mural SMCs is characteristic to ruptured sIA walls, and experiments in animal models suggest that this loss of mural SMCs is causative to sIA growth and eventual rupture [**10**]. The inhibitor of the metalloproteinase, the pathway of nitric oxide and the apoptotic process play a key-role in reducing the resistance of the arterial wall, which can result in the formation and rupture of the intracranial aneurysms [**25**]. Tumor necrosis factor-alpha (TNF-α) has been associated with aneurysms, by inducing phenotypic modulation of cerebral SMCs through myocardin, which demonstrates a novel role for TNF-α in promoting a pro-inflammatory/ matrix-remodeling phenotype, which has important implications for the mechanisms behind IAs formation [**2**]. 

Abnormalities in type III collagen in the arterial walls cause certain familial IAs. The functional variant of COL3A1 is the genetic risk factors for IAs in the Chinese population [**5**]. An association between versican (CSPG2), perlecan (HSPG2), fibrillin 2 (FBN2) and collagen 4A1 (COL4A1) gene variants and IAs have been reported in 2 studies analyzing the Dutch patients. The association of FBN2 and COL4A1 could not be replicated in the Japanese IA population [**30**]. The COL1A2 is located on chromosome 7q22.1, and mutations in this gene have been associated with the development of IAs. The rs2621215 SNP in intron 46 of the COL1A2 gene was found to be marginally associated with an increased risk of IA development in the Korean population [**14**]. 

Type IV collagen α1 and α2 chains form heterotrimers that constitute an essential component of basement membranes. Mutations in COL4A1, encoding the α1 chain, cause a multisystem disease with prominent cerebrovascular manifestations, including porencephaly, bleeding-prone cerebral small vessel disease, and IAs. Therefore, the importance of both COL4A1 and COL4A2 screening was emphasized in patients showing recurrent intracerebral hemorrhage of unknown etiology, particularly if associated with leukoencephalopathy [**13**]. Familial porencephaly, leukoencephalopathy and small-vessel disease belong to the spectrum of disorders credited to dominant mutations in the gene encoding for type IV collagen alpha-1 (COL4A1). Dominant COL4A2 mutations are a novel major risk factor for familial cerebrovascular disease, including porencephaly and small-vessel disease with reduced penetrance and variable phenotype, which might also be modified by other contributing factors [**39**]. Collagen type I α2 (COL1A2) was associated with the presence of aneurysms in patients from Japan, China, and Korea. The COL1A2 gene is associated with IAs in a subset of the German population. However, it is not responsible for the majority of aneurysms, and further candidate genes need to be identified to develop sensitive genetic screening for patients at risk [**12**]. 

## Inflammation, Interleukin, Immunology 

Intracranial aneurysms are acquired lesions (5-10% of the population). In comparison with sporadic aneurysms, familial aneurysms tend to be larger, more often located in the middle cerebral artery, and more likely to be multiple. Other than familiar occurrence, there are several heritable conditions associated with IAs formation, including autosomal dominant polycystic kidney disease, neurofibromatosis type I, Marfan syndrome, multiple endocrine neoplasia type I, pseudoxanthoma elasticum, hereditary hemorrhagic telangiectasia, and Ehlers-Danlos syndrome type II and IV. The familial occurrence and the association with heritable conditions indicate that genetic factors may play a role in the development of IAs [**4**]. Genome-wide linkage studies in families and sib pairs with IAs identified several loci on chromosomes showing suggestive evidence of linkage, particularly on chromosomes 1p34.3-p36.13, 7q11, 19q13.3, and Xp22. A moderate positive association with positional candidate genes was demonstrated (perlecan gene, elastin gene, collagen type 1 A2 gene) for the loci on 1p34.3-p36.13 and 7q11. Moreover, 3 of the polymorphisms analyzed in 2 genes (endothelial nitric oxide synthase T786C, interleukin-6 G572C, and interleukin-6 G174C) were found significantly associated with ruptured/ unruptured aneurysms: the endothelial nitric oxide synthase gene SNPs increased the risk, while IL-6 G174C seemed protective. More recently, two genomic loci (endothelin receptor A and cyclin-dependent kinase inhibitor 2BAS) were found significantly associated with IAs in the Japanese population; endothelin-1 is a potent vasoconstrictor produced by the endothelial cells [**4**].

Interleukin-6 (IL-6) is an important pro-inflammatory cytokine, and some authors demonstrated that IL-6 promoter polymorphism -572G>C is associated with IAs in Caucasian population [**35**] and the IL-6-572GG genotype was associated with a higher risk of IA in a Chinese population [**22**]. The IL-12A and IL-12B independently and jointly are involved in the susceptibility of IA [**20**]. 

Inflammation may also participate in the healing process within IA while playing a protective role against IA rupture [**29**]. Several lines of evidence indicate that inflammatory processes play a key role in the happening and development of IAs. Recently, polymorphisms in the TP53 gene have been shown to be associated with inflammation and inflammatory disease [**19**]. Studies relived a significant association of IA with rs6841581 on chromosome 4q31.23, immediately 5' of the endothelin receptor type A and substantially increased evidence of association for two other regions on chromosomes 12q22 and 20p12.1. This suggests that manipulation of the endothelin pathway may have important implications for the prevention and treatment of IA [**40**]. 

## Conclusions

Genetic factors are responsible for IA formation, especially in multiple and familiarly aneurysms. The affected genes also depend on ethnicity. An important role in aneurysm formation, which correlates with external factors (arterial hypertension, smoke, etc.), is the alteration of the arterial wall because of inflammation. Manipulation of the endothelin pathway may have important implications in the prevention and treatment of IA.
